# A Filmy Black-Phosphorus Polyimide Saturable Absorber for Q-Switched Operation in an Erbium-Doped Fiber Laser

**DOI:** 10.3390/ma9110917

**Published:** 2016-11-11

**Authors:** Tianxian Feng, Dong Mao, Xiaoqi Cui, Mingkun Li, Kun Song, Biqiang Jiang, Hua Lu, Wangmin Quan

**Affiliations:** MOE Key Laboratory of Space Applied Physics and Chemistry, Shaanxi Key Laboratory of Optical Information Technology, School of Science, Northwestern Polytechnical University, Xi’an 710072, China; txfeng@139.com (T.F.); xq_cui@mail.nwpu.edu.cn (X.C.); 2012302868@mail.nwpu.edu.cn (M.L.); songkun@nwpu.edu.cn (K.S.); bqjiang@nwpu.edu.cn (B.J.); hualu@nwpu.edu.cn (H.L.); quanwangmin@nwpu.edu.cn (W.Q.)

**Keywords:** black-phosphorus, Q-switched fiber lasers, saturable absorber

## Abstract

We demonstrate an erbium-doped fiber laser passively Q-switched by a black-phosphorus polyimide film. The multi-layer black-phosphorus (BP) nanosheets were prepared via a liquid exfoliation approach exploiting *N*-methylpyrrolidone as the dispersion liquid. By mixing the BP nanosheets with polyimide (PI), a piece of BP–PI film was obtained after evaporating the mixture in a petri dish. The BP–PI saturable absorber had a modulation depth of 0.47% and was inserted into an erbium-doped fiber laser to realize passive Q-switched operations. The repetition rate of the Q-switched laser increased from 5.73 kHz to 31.07 kHz when the laser pump was enhanced from 31.78 mW to 231.46 mW. Our results show that PI is an excellent host material to protect BP from oxidation, and the BP–PI film can act as a promising nonlinear optical device for laser applications.

## 1. Introduction

Recently, remarkable progress has been made on two-dimensional (2D) nanomaterials including graphene, topological insulators, and transition metal dichalcogenides [1,2,3]. Attributed to their inherent features of high nonlinearity, ultrafast carrier dynamics, and dimensionality effects [4,5], they have found important applications, ranging from ultrafast saturable absorbers (SAs) [6,7,8], photoelectric detectors [9], and field-effect transistors [10] to optical modulators [11]. For the application of ultrafast SAs, graphene exhibits an ultrafast recovery time and broadband optical response, while the weak absorption restricts their light-modulation ability. Topological insulators with insulating bulky states and gapless surface states have been applied as effective optical SAs [12,13,14,15]. Transition metal dichalcogenides (such as MoS_2_ and WS_2_) have been proven to possess thickness-dependent bandgaps from the visible to near-infrared range [16,17,18]. By introducing point defects, the optical response can be extended to longer wavelengths, while the absorption coefficient is still quite small [19].

Black-phosphorus (BP), a newly emerged 2D material, has also attracted interest due to its direct bandgap that is tunable from 1.5 eV to 0.3 eV by reducing the layer number [20,21,22]. Thus, BP is quite attractive at the near and mid-infrared band [23] and fills up the gap between the graphene (zero-bandgap) [24] and the transition metal dichalcogenides (0.7–2 eV) [25,26]. In a single layer, each phosphorus atom is held together with three adjacent atoms by covalent bonds, while different layers are adhered together by the Van der Waals force [27,28], which is similar to that of the graphene [29]. As a result, multi-layer BPs can be obtained using the liquid exfoliation or mechanical cleavage method [30,31,32,33]. Currently, mechanically cleaved BP, microfiber-based multi-layer BP, and BP polymer composites have been used as SAs to generate Q-switched/mode-locked pulses at 1, 1.55, and 2 μm, respectively [21,34,35]. However, in the atmosphere, BP is unstable and tends to be oxidized due to the existence of O_2_ or H_2_O [36,37,38]. The high power illumination induces optical damage that will accelerate the oxidation process. It is quite urgent to develop a flexible, low-cost, BP film to facilitate the application of BP in nonlinear optics and pulsed lasers.

In this paper, we prepare BP nanosheets with a liquid exfoliation approach using *N*-methylpyrrolidone (NMP) as the dispersed liquid. By packaging the nanosheets with the polyimide (PI), the oxidation of the BP can be effectively avoided. After evaporation, a thin BP–PI SA was obtained to realize passive Q-switched operations in an erbium-doped fiber (EDF) laser. By tuning the laser pump from 31.78 mW to 231.46 mW, the pulse repetition rate of the Q-switched laser changed from 5.73 kHz to 31.07 kHz while the pulse duration decreased from 25.77 µs to 3.59 µs.

## 2. Methods

**Figure 1:**
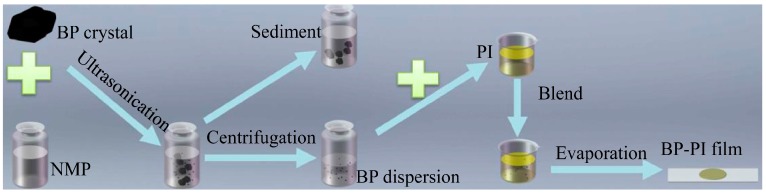
Schematic diagram for preparing BP–PI films. NMP: *N*-methylpyrrolidone; black-phosphorus: BP; polyimide: PI.

Several approaches have been proposed to fabricate multi- and few-layer 2D materials, such as liquid exfoliation, chemical vapor deposition, and mechanical cleavage [39,40]. Each method has its own advantages and application fields. Among them, the liquid exfoliation method is a simple but quite effective technique for preparing low-dimensional nanomaterials from their bulk crystals under an ambient atmosphere [19,41,42,43]. Herein, the multi-layer BP nanosheets are also prepared via the liquid exfoliation method, as depicted in Figure 1. First, the bulk-state BP crystal (50 mg) is added into an NMP solution (30 mL), and the mixture is then bath-sonicated at 180 W for 4 h with a cell crusher. Second, the dispersion of the BP nanosheets is centrifuged for 5 min at a speed of 500 rpm to remove the unwanted large BP sediment. After that, the upper supernatant BP dispersion is collected to prepare the sample. Third, by mixing the PI with BP nanosheets, a thin BP–PI film can be obtained by evaporating the dispersion on a petri dish.

**Figure 2:**
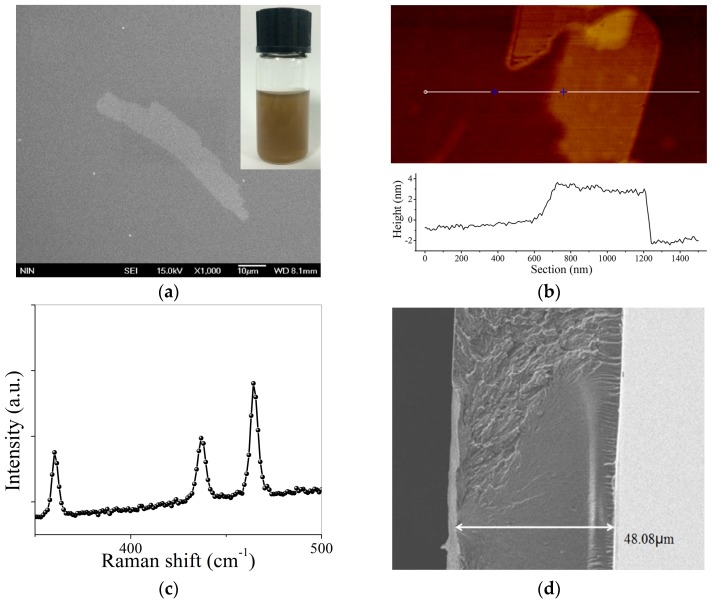
Characterization of BP nanosheets and BP–PI films. (**a**) Scanning electron microscope (SEM) image of the BP nanosheets. The inset is the photograph of the BP nanosheets dispersion; (**b**) atomic force microscopy (AFM) image of the BP nanosheets; (**c**) Raman spectrum of the BP sample; (**d**) sectional view of the BP–PI film.

As shown in the inset of Figure 2a, the BP suspension exhibits a faint brown color and is quite stable over dozens of days. The scanning electron microscope (SEM) image in Figure 2a illustrates that the width and length of the as-prepared BP nanosheets range from 0.5 µm to 10 µm. It is closely related to the centrifugation rate in the fabrication process. As shown in Figure 2b, the thickness of most BP nanosheets is given as ~4 nm from the atomic force microscopy (AFM) result. The thickness single layer BP is ~0.6 nm [21], which implies that the as-prepared samples are multi-layer BPs. The BP sample is further identified from the Raman spectrum. As depicted in Figure 2c, three peaks at 360.2 cm^−1^, 436.9 cm^−1^, and 464.3 cm^−1^ that correspond to $A_{g}^{1}$, B_2g_, and $A_{g}^{2}$ vibration modes of the BP, respectively, are clearly observed on the Raman spectrum. The side profile of the BP–PI film is shown in Figure 2d, which shows a thickness of 48.08 µm. The experimental results indicate that BP nanosheets and the BP–PI film was successfully prepared using the proposed method.

## 3. Results and Discussion

**Figure 3:**
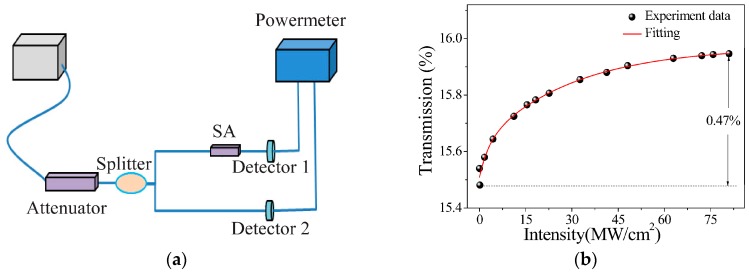
(**a**) Scheme of the balanced twin-detector system; (**b**) nonlinear transmission of BP–PI saturable absorber (SA).

The saturable absorption property of the BP–PI film was measured by a balanced twin-detector technique, as described in Figure 3a. The illumination pulse was generated from a passively mode-locked fiber laser, and the optical intensity was adjusted by an attenuator. After that, the pulse was split equally with a fiber coupler, in which one branch worked as a reference beam and the other branch was inserted with the prepared sample. By comparing the pulse intensities of the two branches, the transmission of the film versus the pulse intensity was obtained [44]. As depicted in Figure 3b, the BP–PI film exhibited typical characteristics of an SA in that the transmission increased with pulse intensity. Based on the fitting results, the modulation depth of the BP–PI film was 0.47%.

**Figure 4:**
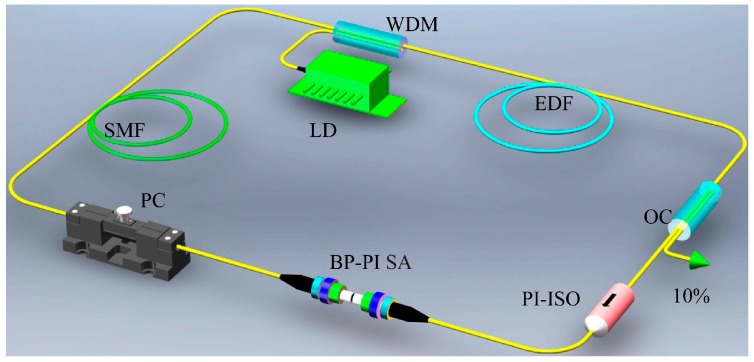
Experiment setup of the EDF laser Q-switched by a BP–PI SA. LD: laser diode; WDM: wavelength division multiplexer; PI-ISO: polarization-independent isolator; EDF: erbium-doped fiber; SMF: single-mode fiber; OC: optical coupler; BP–PI SA: black-phosphorus–polyimide saturable absorber; PC: polarization controller.

Figure 4 is a sketch map of the EDF laser Q-switched by a BP–PI SA. The laser resonator is composed of an optical coupler, wavelength division multiplexer, a 6-m-long EDF, a polarization controller, a polarization-independent isolator, and a BP–PI SA. The EDF with an absorption coefficient of 3 dB/m acts as the gain medium in the laser and a 980 nm laser diode pumps the EDF through the wavelength division multiplexer. The 10% optical coupler is used to output the laser emission, and the isolator forces the laser to operate at a unidirectional state. The polarization controller is used for tuning the polarization state inside the laser cavity, and the obtained BP–PI film is transferred into the optical fiber ferrules to prepare the fiber-based SA. The other fibers and pigtails of the devices are standard single mode fibers that have a total length of 33 m.

**Figure 5:**
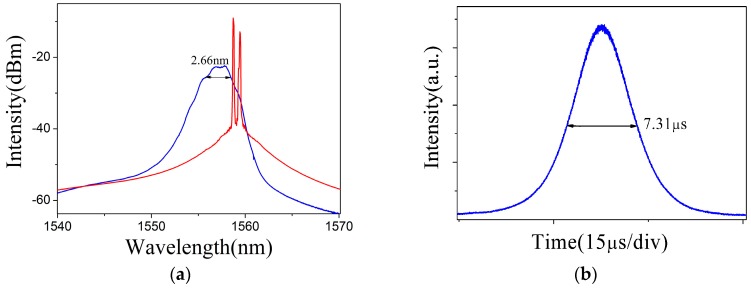

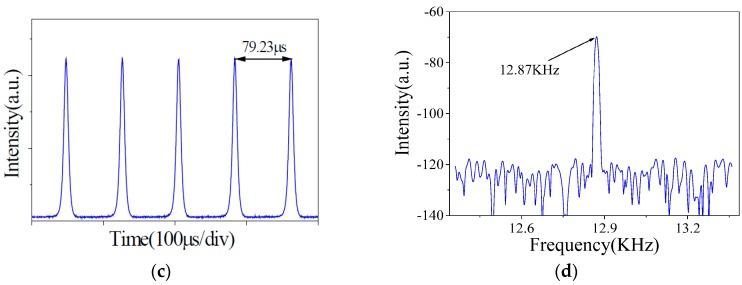
(**a**) Output spectrum; (**b**) pulse profile; (**c**) pulse train; and (**d**) radio-frequency spectrum of the Q-switched fiber laser at the pump of 93.94 mW.

When the pump power reached 25.07 mW, a continuous laser was obtained without inserting the BP–PI film in the resonator, as the red curve shows in Figure 5a. After inserting the BP–PI SA, self-started stable Q-switched pulses were observed in the fiber laser when the laser pump approached 31.78 mW. A typical Q-switched operation at the laser pump of 93.94 mW is plotted in Figure 5. As shown in Figure 5a, the laser spectrum was centered at 1556.93 nm and the 3 dB spectral width was given as 2.66 nm. The pulse profile and typical pulse train are shown in Figure 5b,c, which shows a pulse duration of 7.31 μs and a pulse interval of 79.23 μs, respectively. The radio-frequency spectrum in Figure 5d shows that the pulse operated at a repetition rate of 12.87 KHz, in agreement with the pulse–pulse interval.

**Figure 6:**
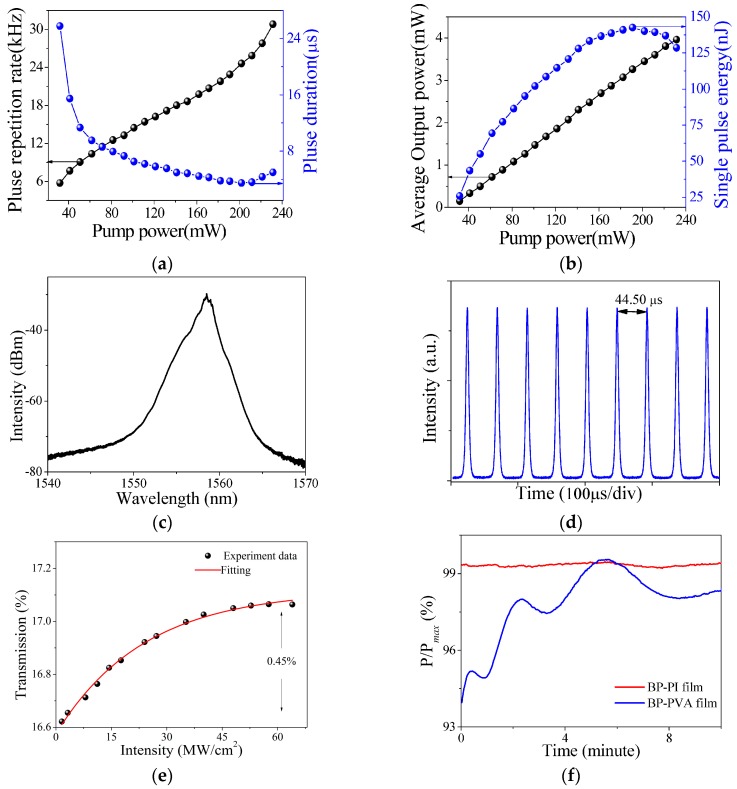
(**a**) Repetition rate, pulse duration as well as (**b**) average output power, single-pulse energy of Q-switched pulses at different pump powers; (**c**) optical spectrum and (**d**) pulse train of the Q-switched laser using the BP–PI film after stored for six months; (**e**) saturable absorption property of the film; (**f**) stability of the BP–PI film and the BP–PVA film versus the laser operation time.

A typical characteristic of Q-switched pulses is that the repetition rate as well as the pulse duration changes with pump powers, as demonstrated in Figure 6a. For example, by varying the laser pump from 31.87 mW to 231.46 mW, the repetition rate changed from 5.73 kHz to 31.07 kHz and the duration was reduced from 25.77 µs to 3.59 µs. The output power as well as single-pulse energy at different pump powers are illustrated in Figure 6b. With the enhancement of the laser pump, the average output power almost rose linearly to 4.2 mW. Correspondingly, the pulse energy increased firstly to 142.60 nJ and then decreased 128.21 nJ when the laser pump was higher than 191.04 mW. Further increasing the laser pump, an unstable transitional state between mode-locking and Q-switching was found in the fiber laser. By removing the BP–PI film or replacing the BP–PI film with a pure PI film, Q-switched pulses disappeared immediately in the fiber laser, even though the polarization controller or the pump power was tuned over a full range tens of times. Moreover, the Q-switched state could be established again by inserting the BP–PI film into the fiber laser, which indicates that the BP nanosheets played a key role in the shaping of the Q-switched pulse. However, the stable mode locking operation was not observed in the fiber laser with the proposed SA. These results may be attributed to the large inset loss or small modulation depth of the SA. For example, when the dispersion-induced temporal broadening or the output-induced perturbation could not be compensated by the SA, the mode-locking operation will not be established in the fiber laser.

The BP–PI film exhibits two obvious advantages. First, the Q-switched operation can still be realized when the BP–PI film is stored for six months or longer in air. Figure 6c,d show the optical spectrum and pulse train of the Q-switched laser using the BP–PI film from the same sample. However, Q-switched operation cannot be achieved again using a BP–polyvinyl alcohol (PVA) film. This result may be attributed to the highly hydrophilic nature of the PVA. Figure 6e shows that the BP–PI film still exhibits a saturable absorption property after six months or more. Second, we compared the stability of the BP–PI film and the BP–PVA film versus the laser operation time. In the experiment, the BP–PI film and the BP–PVA film were inserted into the fiber laser, severally. The output powers were recorded by a power meter for 10 min. After being divided by the maximum of the output power, the stability of the BP–PI film and the BP–PVA film versus the laser operation time can be obtained, as shown in Figure 6f. One can observe that the output power had a larger disturbance for the BP–PVA film than that of the BP–PI film, which may be attributed to the different fusing points of PI (450 °C) and PVA (180 °C). For instance, the laser-induced heat accumulation destroys the BP–PVA film more easily. The PI is capable of protecting BP from intense laser illumination without oxidation, which is very important in the applications of ultrafast lasers and nonlinear optics.

## 4. Conclusions

We fabricated a BP–PI SA to achieve the passive Q-switched operation in an EDF laser. The BP nanosheets were prepared via the liquid exfoliation approach utilizing NMP as the dispersion solvent. The BP–PI SA was obtained by mixing the PI with BP nanosheets and then evaporating the mixture. Based on the proposed BP–PI SA, the fiber laser delivered the Q-switched pulse with the maximum repetition rate, pulse energy, and minimum pulse duration of 31.07 kHz, 142.60 nJ, and 3.59 µs respectively. These results clearly show that a BP–PI film can be an excellent SA for Q-switched fiber lasers, and may find further applications in such areas as frequency conversion and optical limiting.

## References

[1] Bonaccorso F., Sun Z., Hasan T., Ferrari A. (2010). Graphene photonics and optoelectronics. Nat. Photonics.

[2] Eda G., Maier S.A. (2013). Two-dimensional crystals: Managing light for optoelectronics. ACS Nano.

[3] Mohanraj J., Velmurugan V., Sivabalan S. (2016). Transition metal dichalcogenides based saturable absorbers for pulsed laser technology. Opt. Mater..

[4] Dawlaty J.M., Shivaraman S., Chandrashekhar M., Rana F., Spencer M.G. (2008). Measurement of ultrafast carrier dynamics in epitaxial graphene. Appl. Phys. Lett..

[5] Mak K.F., Sfeir M.Y., Wu Y., Lui C.H., Misewich J.A., Heinz T.F. (2008). Measurement of the optical conductivity of graphene. Phys. Rev. Lett..

[6] Zhang H., Bao Q., Tang D., Zhao L., Loh K. (2009). Large energy soliton erbium-doped fiber laser with a graphene-polymer composite mode locker. Appl. Phys. Lett..

[7] Zhao C., Zhang H., Qi X., Chen Y., Wang Z., Wen S., Tang D. (2012). Ultra-short pulse generation by a topological insulator based saturable absorber. Appl. Phys. Lett..

[8] Mao D., Wang Y., Ma C., Han L., Jiang B., Gan X., Hua S., Zhang W., Mei T., Zhao J. (2015). WS_2_ mode-locked ultrafast fiber laser. Sci. Rep..

[9] Xia F., Mueller T., Lin Y.-M., Valdes-Garcia A., Avouris P. (2009). Ultrafast graphene photodetector. Nat. Nanotechnol..

[10] Radisavljevic B., Radenovic A., Brivio J., Giacometti I.V., Kis A. (2011). Single-layer MoS_2_ transistors. Nat. Nanotechnol..

[11] Li W., Chen B., Meng C., Fang W., Xiao Y., Li X., Hu Z., Xu Y., Tong L., Wang H. (2014). Ultrafast all-optical graphene modulator. Nano Lett..

[12] Chen Y., Analytis J., Chu J.-H., Liu Z., Mo S.-K., Qi X.-L., Zhang H., Lu D., Dai X., Fang Z. (2009). Experimental realization of a three-dimensional topological insulator, Bi_2_Te_3_. Science.

[13] Jung M., Lee J., Koo J., Park J., Song Y.-W., Lee K., Lee S., Lee J.H. (2014). A femtosecond pulse fiber laser at 1935 nm using a bulk-structured Bi_2_Te_3_ topological insulator. Opt. Express.

[14] Mao D., Jiang B., Gan X., Ma C., Chen Y., Zhao C., Zhang H., Zheng J., Zhao J. (2015). Soliton fiber laser mode locked with two types of film-based Bi_2_Te_3_ saturable absorbers. Photonics Res..

[15] Chen Y., Zhao C., Huang H., Chen S., Tang P., Wang Z., Lu S., Zhang H., Wen S., Tang D. (2013). Self-Assembled Topological Insulator: Bi_2_Se_3_ Membrane as a Passive Q-Switcher in an Erbium-Doped Fiber Laser. J. Lightwave Technol..

[16] Wang Q.H., Kalantar-Zadeh K., Kis A., Coleman J.N., Strano M.S. (2012). Electronics and optoelectronics of two-dimensional transition metal dichalcogenides. Nat. Nanotechnol..

[17] Mao D., Du B., Yang D., Zhang S., Wang Y., Zhang W., She X., Cheng H., Zeng H., Zhao J. (2016). Nonlinear Saturable Absorption of Liquid–Exfoliated Molybdenum/Tungsten Ditelluride Nanosheets. Small.

[18] Guo B., Yao Y., Xiao J.-J., Wang R.-L., Zhang J.-Y. (2016). Topological insulator-assisted dual-wavelength fiber laser delivering versatile pulse patterns. IEEE. J. Sel. Top. Quant..

[19] Mao D., Zhang S., Wang Y., Gan X., Zhang W., Mei T., Wang Y., Wang Y., Zeng H., Zhao J. (2015). WS_2_ saturable absorber for dissipative soliton mode locking at 1.06 and 1.55 µm. Opt. Express.

[20] Li L., Yu Y., Ye G.J., Ge Q., Ou X., Wu H., Feng D., Chen X.H., Zhang Y. (2014). Black phosphorus field-effect transistors. Nat. Nanotechnol..

[21] Luo Z.-C., Liu M., Guo Z.-N., Jiang X.-F., Luo A.-P., Zhao C.-J., Yu X.-F., Xu W.-C., Zhang H. (2015). Microfiber-based few-layer black phosphorus saturable absorber for ultra-fast fiber laser. Opt. Express.

[22] Wang Z., Xu Y., Dhanabalan S.C., Sophia J., Zhao C., Xu C., Xiang Y., Li J., Zhang H. (2016). Black Phosphorus Quantum Dots as an Efficient Saturable Absorber for Bound Soliton Operation in an Erbium Doped Fiber Laser. IEEE Photonics J..

[23] Sotor J., Sobon G., Macherzynski W., Paletko P., Abramski K.M. (2015). Black phosphorus saturable absorber for ultrashort pulse generation. Appl. Phys. Lett..

[24] Wang Z., Zou Y., Chen Y., Wu M., Zhao C., Zhang H., Wen S. (2013). Graphene sheet stacks for Q-switching operation of an erbium-doped fiber laser. Laser Phys. Lett..

[25] Berkdemir A., Gutiérrez H.R., Botello-Méndez A.R., Perea-López N., Elías A.L., Chia C.-I., Wang B., Crespi V.H., López-Urías F., Charlier J.-C. (2013). Identification of individual and few layers of WS_2_ using Raman Spectroscopy. Sci. Rep..

[26] Mu H., Lin S., Wang Z., Xiao S., Li P., Chen Y., Zhang H., Bao H., Lau S.P., Pan C. (2015). Black phosphorus-polymer composites for pulsed lasers. Adv. Opt. Mater..

[27] Liu H., Du Y., Deng Y., Peide D.Y. (2015). Semiconducting black phosphorus: Synthesis, transport properties and electronic applications. Chem. Soc. Rev..

[28] Al-Masoodi A., Ahmed M., Latiff A., Arof H., Harun S. (2016). Q-Switched Ytterbium-Doped Fiber Laser Using Black Phosphorus as Saturable Absorber. Chin. Phys. Lett..

[29] Geim A.K., Grigorieva I.V. (2013). Van der Waals heterostructures. Nature.

[30] Wang Y., Huang G., Mu H., Lin S., Chen J., Xiao S., Bao Q., He J. (2015). Ultrafast recovery time and broadband saturable absorption properties of black phosphorus suspension. Appl. Phys. Lett..

[31] Xu Y., Wang Z., Guo Z., Huang H., Xiao Q., Zhang H., Yu X.-F. (2016). Solvothermal synthesis and ultrafast photonics of black phosphorus quantum dots. Adv. Opt. Mater..

[32] Li D., Jussila H., Karvonen L., Ye G., Lipsanen H., Chen X., Sun Z. (2015). Polarization and thickness dependent absorption properties of black phosphorus: New saturable absorber for ultrafast pulse generation. Sci. Rep..

[33] Xia F., Wang H., Jia Y. (2014). Rediscovering black phosphorus as an anisotropic layered material for optoelectronics and electronics. Nat. Commun..

[34] Chen Y., Jiang G., Chen S., Guo Z., Yu X., Zhao C., Zhang H., Bao Q., Wen S., Tang D. (2015). Mechanically exfoliated black phosphorus as a new saturable absorber for both Q-switching and Mode-locking laser operation. Opt. Express.

[35] Hisyam M., Rusdi M., Latiff A., Harun S. (2016). Generation of Mode-locked Ytterbium doped fiber ring laser using few-layer black phosphorus as a saturable absorber. IEEE J. Sel. Top. Quantum Electron..

[36] Wood J.D., Wells S.A., Jariwala D., Chen K.S., Cho E., Sangwan V.K., Liu X., Lauhon L.J., Marks T.J., Hersam M.C. (2014). Effective passivation of exfoliated black phosphorus transistors against ambient degradation. Nano Lett..

[37] Andres C.-G., Leonardo V., Elsa P., Joshua O.I., Narasimha-Acharya K.L., Sofya I.B., Dirk J.G., Michele B., Gary A.S., Alvarez J.V. (2014). Isolation and characterization of few-layer black phosphorus. 2D Mater..

[38] Pieter J.D.V., Rebekah C., Joshua O.I., Matvey F., Allard J.K., Holger T., Herre S.J.V.D.Z., Teun M.K. (2016). Spatial conductivity mapping of unprotected and capped black phosphorus using microwave microscopy. 2D Mater..

[39] Huang X., Zeng Z., Zhang H. (2013). Metal dichalcogenide nanosheets: Preparation, properties and applications. Chem. Soc. Rev..

[40] Island J.O., Steele G.A., van der Zant H.S., Castellanos-Gomez A. (2015). Environmental instability of few-layer black phosphorus. 2D Mater..

[41] Coleman J.N., Lotya M., O’Neill A., Bergin S.D., King P.J., Khan U., Young K., Gaucher A., De S., Smith R.J. (2011). Two-dimensional nanosheets produced by liquid exfoliation of layered materials. Science.

[42] Brent J.R., Savjani N., Lewis E.A., Haigh S.J., Lewis D.J., O’Brien P. (2014). Production of few-layer phosphorene by liquid exfoliation of black phosphorus. Chem. Commun..

[43] Mao D., She X., Du B., Yang D., Zhang W., Song K., Cui X., Jiang B., Peng T., Zhao J. (2016). Erbium-doped fiber laser passively mode locked with few-layer WSe_2_/MoSe_2_ nanosheets. Sci. Rep..

[44] Ahmad H., Zulkifli A.Z., Muhammad F.D., Zulkifli M.Z., Thambiratnam K., Harun S.W. (2013). Mode-locked L-band bismuth–erbium fiber laser using carbon nanotubes. Appl. Phys. B.

